# Production of recombinant IgA1 with defined mucin-type O-glycans in *Nicotiana tabacum* BY-2 cells

**DOI:** 10.3389/fpls.2025.1675517

**Published:** 2025-09-24

**Authors:** Nicolas Bailly, Clemens Grünwald-Gruber, Marie Peeters, François Chaumont, Catherine Navarre

**Affiliations:** ^1^ Louvain Institute of Biomolecular Science and Technology (LIBST), UCLouvain, Louvain-la-Neuve, Belgium; ^2^ Core Facility Mass Spectrometry, University of Natural Resources and Life Sciences, Vienna, Austria

**Keywords:** protein glycosylation, O-glycosylation, IgA1, *Nicotiana tabacum* BY-2 cells, recombinant glycoprotein

## Abstract

*Nicotiana tabacum* BY-2 cell suspension cultures are a powerful platform for producing recombinant glycoproteins such as immunoglobulins. Extensive efforts have been devoted to engineering the N-glycosylation pathway of BY-2 cells to overcome differences between mammalian and plant N-glycans. However, the mucin-type O-glycosylation pathway is absent in plant cells. This modification, which consists of glycan attachment to serine or threonine residues, is important in many human proteins, but is also highly complex. In this regard, plants offer a unique opportunity to engineer this pathway *de novo* without the interplay of many competing enzymes. In this study, transgenic BY-2 cell lines expressing the enzymes responsible for the formation of Core 1 and Tn antigen glycans were generated. First, GalNAc-O-glycosylation was initiated by the expression of human GalNAcT2. This O-glycan was then elongated by co-expression of Drosophila C1GalT1 to form the core 1 structure. Human IgA1 was produced in these engineered BY-2 cell lines and the presence of mucin-type O-glycans was confirmed by lectin blotting. The precise O-glycosylation profile of the hinge region was determined by mass spectrometry and showed the almost complete disappearance of pentoses and the presence of core 1 O-glycans.

## 1 Introduction

Suspension cultures of plant cells, particularly *Nicotiana tabacum* BY-2 cells, are an interesting platform for the production of biopharmaceuticals (Santos et al., 2016; Karki et al., 2021; Xu et al., 2025). This system combines the cost effectiveness and safety of plant cells with the industry GMP compliance, and some human enzymes produced in plant suspension cultures have been approved by regulatory agencies (Elelyso^®^ and Elfabrio^®^). Therapeutic proteins are often glycosylated, and this modification has a critical impact on their properties. N-glycoengineering in BY-2 cells has already been well documented. In particular, the removal of non-human core alpha(1,3)-fucose and beta(1,2)-xylose structures has been successfully achieved in BY-2 cells by edition of alpha(1,3)-fucosyltranferase and beta(1,2)-xylosyltransferase genes by CRISPR/Cas9 (Hanania et al., 2017; Mercx et al., 2017; Sheva et al., 2020), allowing the production of glycoproteins with human-like complex N-glycans in *XylT*/*FucT*-KO (ΔXT/FT) BY-2 cells.

In humans, GalNAc-O-glycosylation on the oxygen atom of serine and threonine, also known as mucin-type, is the most abundant type of O-glycosylation, but this O-glycosylation pathway is absent in plants. The initiation of mucin-type GalNAc-O-glycosylation has been successfully demonstrated in plants. The expression of human GalNAcT2 (or GalNAcT4) in *Nicotiana benthamiana* leaves or transgenic BY-2 cells is functional on relevant glycoproteins such as MUC1, MUC16, interferon alpha 2b, EPO or G-CSF (Yang et al., 2012b, Yang et al., 2012a; Castilho et al., 2012; Ramírez-Alanis et al., 2018; Daskalova et al., 2010). The elongation of the GalNAc-O glycan in core 1 O-glycan structure has also been established for EPO produced *in N. benthamiana* leaves by co-expression of *Drosophila melanogaster* C1GalT1 (Castilho et al., 2012). Another important O-glycosylated protein is human IgA1, which displays a long hinge region with three to six O-glycan chains attached to some of the nine potential threonine/serine O-glycosylation sites (Ohyama et al., 2020). A few studies have described the successful engineering of IgA1 hinge region O-glycosylation upon co-expression of enzymes from the mucin O-glycosylation machinery in wild type or ΔXT/FT *N. benthamiana* leaves (Uetz et al., 2024; Dicker et al., 2016).

In this study, we generated a transgenic *N. tabacum* ΔXT/FT BY-2 cell line capable of producing Tn antigen or core 1 mucin-type O-glycans. Coexpression of human GalNAcT2 with Drosophila C1GalT in BY-2 cells resulted in the production of a human IgA1 variant of Trastuzumab with a hinge region displaying up to five core 1 O-glycan structures.

## 2 Material and methods

### 2.1 Cloning and expression vectors

The genetic constructs were obtained using the Golden Gate plant toolkit (Engler et al., 2014) following the protocol described by (Marillonnet and Grützner, 2020). The cloning of the level 0 plasmids pICH41258-SPPDI, pICH41295-pPMA4, pGEMT-tPMP1 and pICH41331-SAR and the level 1 pICH47732-nptII and pICH47732-hptI is described in (Navarre et al., 2023).The ORFs encoding human GalNAcT2 (NP_004472.1) and *D. melanogaster* C1GalT1 (NP_609258.1) were designed to be introduced into the level 0 CDS1 Golden Gate plasmid (pICH41308): AATG and GCTT fusion sites were added with BsaI restriction sites in 5’ and 3’, respectively. The sequences were synthesized by Genewiz (GalNAcT2) and Genscript (C1GalT1) and cloned into pICH41308. Next, expression cassettes were constructed into level 1 plasmids under the control of p35S promoter (pICH51288) and tNOS terminator (pICH41421). Different level 1 plasmids were used to define the correct position of the expression cassettes in the final constructs: pICH47742 (position 2, GalNAcT2 or C1GalT1) and pICH47751 (position 3, C1GalT1). In addition, the neomycin phosphotransferase II expression cassette from the kit (pICSL70004) was transferred in the plasmid pICH47732 at position 1. Finally, the genetic insulator of Rb7 scaffold attachment region (SAR) of *N. tabacum* (1.2 kb), which should reduce positional and silencing effects (Diamos and Mason, 2018), was transferred into level 1 at position 3 (pICH47751), 4 (pICH47761) or 5 (pICH47772). The expression cassettes were then assembled into the level M pAGM8031 plasmid to generate pAGM8031-nptII-GalNAcT2-SAR and pAGM8031-nptII-GalNAcT2-C1GalT1-SAR.

Sequences coding for IgA1 HC and LC were provided by Strasser’s laboratory in pEAQ (Göritzer et al., 2017) and PCR-amplified to add 5’-AATG and GCTT-3’ and BpiI restriction sites for cloning into the level 0 CDS1 plasmid pICH41308. The nucleotide sequence corresponding to the intronless *N. tabacum* Extensin terminator (tExt) was PCR amplified from pBYR2eKMd-GFP (Diamos and Mason, 2018) to add 5’-GCTT and CGCT-3’ and BsaI restriction sites and cloned into pICH41276. The ORFs were then assembled with pPMA4 and tExt in different level 1 plasmids: pICH47742 and pICH47761 for HC; pICH47751 and pICH47772 for LC. The HC and LC expression cassettes were then assembled with the nptII, GalNAcT2 and C1GalT1 expression cassettes into the level M plasmid pAGM8031 to generate: pAGM8031-nptII-HC-LC, pAGM8031-nptII-HC-LC-GalNAcT2 and pAGM8031-nptII-GalNAcT2-C1GalT1-HC-LC.

### 2.2 Transformation of BY-2 cells

*Agrobacterium tumefaciens* LBA4404 VirG-mediated stable transformation of ΔXT/FT BY-2 cells was carried out as described by (Navarre and Chaumont, 2022) with *npt*II resistance marker gene. Selection of transformed cell lines was carried out on MS-agar plates containing 500 µg/mL cefotaxime, 400 µg/mL carbenicillin and 100 µg/mL kanamycin.

Samples used for western blotting were harvested from liquid MS cultures obtained after at least two passages on solid selective medium (1–2 months) and at least two passages in liquid selective medium. D11b cultures (Vasilev et al., 2013) were grown for ten days in 50 mL of medium without cyclodextrin, in 250 mL Erlenmeyer flasks.

### 2.3 IgA1 protein purification

IgA1 purification was adapted from the protocol described by (Mercx et al., 2017). Briefly, 50–250 mL of 10-day-old BY-2 suspension culture grown in D11b medium (Vasilev et al., 2013) without cyclodextrin was filtered on three layers of Miracloth and the filtrate was centrifuged (13,900 *g*, 30 min). The supernatant was recovered, supplemented with 10% 1 M Tris-Cl pH 8.0 and incubated for 16 h at 4 °C with 400 µL of Pierce R Protein A Plus Agarose (Thermoscientific # 22812) previously washed three times with 4 mL of 0.1 M Tris-Cl pH 8.0. The sample was centrifuged at ~50 *g* for 5 min and the supernatant was discarded. The beads were washed twice with 10 mL of 0.1 M Tris-Cl pH 8.0 and transferred into a 10 mL Poly-Prep chromatography column (Bio-Rad #7311550). IgA1 was eluted with 5 × 500 µL 0.1 M glycine-HCl pH 3.0 and immediately buffered with 10% 1 M Tris-HCl pH 8.0.

### 2.4 Protein extraction

Two mL of the indicated BY-2 cultures were filtered through three layers of Miracloth by centrifugation (2,900 *g* for 5 min). The filtered BY-2 cells were used to harvest cellular total soluble proteins (TSP) and microsomal fractions (MF). The cell packs were transferred into a 2-mL screw cap microtube containing 0.5 glass beads (0.85-1.23 mm) and frozen in liquid nitrogen. Next, 700 µL of homogenization buffer (250 mM sorbitol, 2 mM Na_2_EDTA, 60 mM Tris-HCl pH 8.0) supplemented with 1 mM PMSF and protease inhibitor cocktail (leupeptin, aprotinin, antipain, pepstatin and chymostatin, each at 2 µg/mL) were added. The cells were ground for 3 x 40 s at 5,000 rpm (Precellys 24 tissue homogenizer, Bertin Technologies) with 2 min pauses on ice. The samples were first centrifuged for 5 min at 2,800 *g* at 4 °C, then for 7 min at 10,000 *g* at 4 °C. To separate TSP from the MF, the samples were centrifuged for 15 min at 130,000 *g* at 4 °C. The supernatant (TSP) was collected and the pellet (MF), was resuspended by sonication in 50 µL resuspension buffer (3 mM KH_2_PO_4_, 330 mM sucrose, 3 mM KCl, pH 7.8 (KOH)). Protein concentrations were determined according to Bradford (Bradford, 1976) using BSA as standard.

### 2.5 SDS-PAGE, western blotting and lectin blotting

The indicated amount of TSP, MF or purified IgA1 was analyzed by reducing SDS-PAGE after solubilization (5 min at 100 °C for TSP or purified IgA1; 15 min at 56 °C for MF). For non-reducing SDS-PAGE, purified IgA1 samples were solubilized for 30 min at room temperature. Gels were stained with Coomassie Blue G-250 (SERVA, 17524) or transferred onto PVDF membrane (Bio-Rad laboratories).

For western blotting, the membranes were saturated into Tris Buffer Saline containing 0.5% Tween^®^20 (TBST) and 3% (w/v) milk powder, before incubation with rabbit polyclonal antibodies against hGalNAcT2 (Sino Biologicals, 13764-T62, 1:2,000) and anti-rabbit HRP polyclonal antibodies (SynAbs, LO-RG-1-HRP, 1:10,000), or goat polyclonal antibodies against human IgA (alpha heavy chain) couple to HRP (Invitrogen, 31417, 1:25,000).

For lectin blotting, the PVDF membranes were saturated in TBST containing 2% (w/v) BSA before incubation with biotinylated *Helix pomatia* agglutinin (Sigma-Aldrich L6512, 1:2,500) or biotinylated peanut agglutinin (Vector Laboratories B1075, 1:2,500), followed by incubation with Streptavidin HRP (Roche 11089153001, 1:5,000).

Chemiluminescence was detected after revelation with BM chemilumiscence blotting substrate (Roche 11500694001) using Amersham Imager 600 (GE Healthcare).

### 2.6 *In vitro* deglycosylation

Purified IgA1 protein (125 ng) was denatured by heating for 10 min at 100 °C in the manufacturer’s glycoprotein denaturing buffer to ensure optimal glycosite accessibility. Samples were then incubated for 1 h at 37 °C with 50 U of EndoH (New England Biolabs P0703) or PNGase F (New England Biolabs P0704). Control samples were run in parallel, but without the glycosidase. The samples were then visualized by SDS-PAGE followed by western blotting using goat polyclonal antibodies raised against human IgA (alpha heavy chain) couple to HRP (Invitrogen 31417, 1:25,000) and revealed with BM chemilumiscence blotting substrate (Roche 11500694001). Chemiluminescence was detected using the Amersham Imager 600 (GE Healthcare).

### 2.7 Thermal shift assay

The thermal stability of the three Trastuzumab IgA1 O-glycovariants was analyzed by a different scanning fluorimetry assay following the protocol described by (Herman et al., 2023). Briefly, purified IgA1 samples (1.2 µg) were diluted twice in phosphate buffered saline (100 mM phosphate, 150 mM NaCl, pH 7.0) containing 5x SYPRO Orange Protein Gel Stain (S6650; Invitrogen, Waltham, MA, USA). The samples were transferred into a MicroAmpTM Optical 96-Well Reaction Plate (N8010560; Applied Biosystems, Waltham, MA, USA). Each sample was run as a technical triplicate. DSF measurements were performed using a temperature increase from 15 °C to 85 °C with a ramp of 1 °C/min (StepOnePlusTM Real-Time PCR System; Applied Biosystems). The mean melting temperatures were calculated as the temperature corresponding to the maximum value of the mean derivative curves. Mean derivative curves were obtained as the first derivative of the mean melt curves.

### 2.8 Mass spectrometry analysis IgA1 glycopeptides

The IgA1 O-glycovariants were digested in-solution. The proteins were S-alkylated with iodoacetamide and digested with trypsin (Promega) overnight. The peptide samples were then digested with GluC (Promega) for 4 h. The digested samples were loaded on a nanoEase C18 column (nanoEase M/Z HSS T3 Column, 100Å, 1.8 μm, 300 μm X 150 mm, Waters) using 0.1% formic acid as the aqueous solvent. A gradient from 1% B (B: 80% acetonitrile, 0.1% formic acid) to 40% B in 50 min was applied, followed by a 5 min gradient from 40% B to 95% B that facilitates elution of large peptides, at a flow rate of 6 μL/min. Detection was performed with an Orbitap MS (Exploris 480, Thermo) equipped with the standard H-ESI source in positive ion, DDA mode (= switching to MSMS mode for eluting peaks). MS scans were recorded (range: 350–3200 m/z) and the 8 highest peaks were selected for fragmentation. Instrument calibration was performed using Pierce FlexMix Calibration Solution (Thermo Scientific). The possible glycopeptides were identified as sets of peaks consisting of the peptide moiety and the attached N-glycan varying in the number of N-acetylhexosamine (HexNAc) units, hexose (Hex), pentose (Pent) and deoxyhexose (dHex) residues, as well as additional hydroxyproline modifications for the O-glycosylation site. The theoretical masses of these glycopeptides were determined with a spread sheet using the monoisotopic masses for amino acids and monosaccharides. Manual glycopeptide searches were made using FreeStyle 1.8 (Thermo). For the relative quantification of the different glycoforms, the peak intensities of the deconvoluted spectra were compared (using an in-house software tool. (Freestyle_parser_v0.3.R)).

## 3 Results

### 3.1 Stable engineering of mucin-type O-glycosylation in humanized BY-2 cells

**Figure 1:**
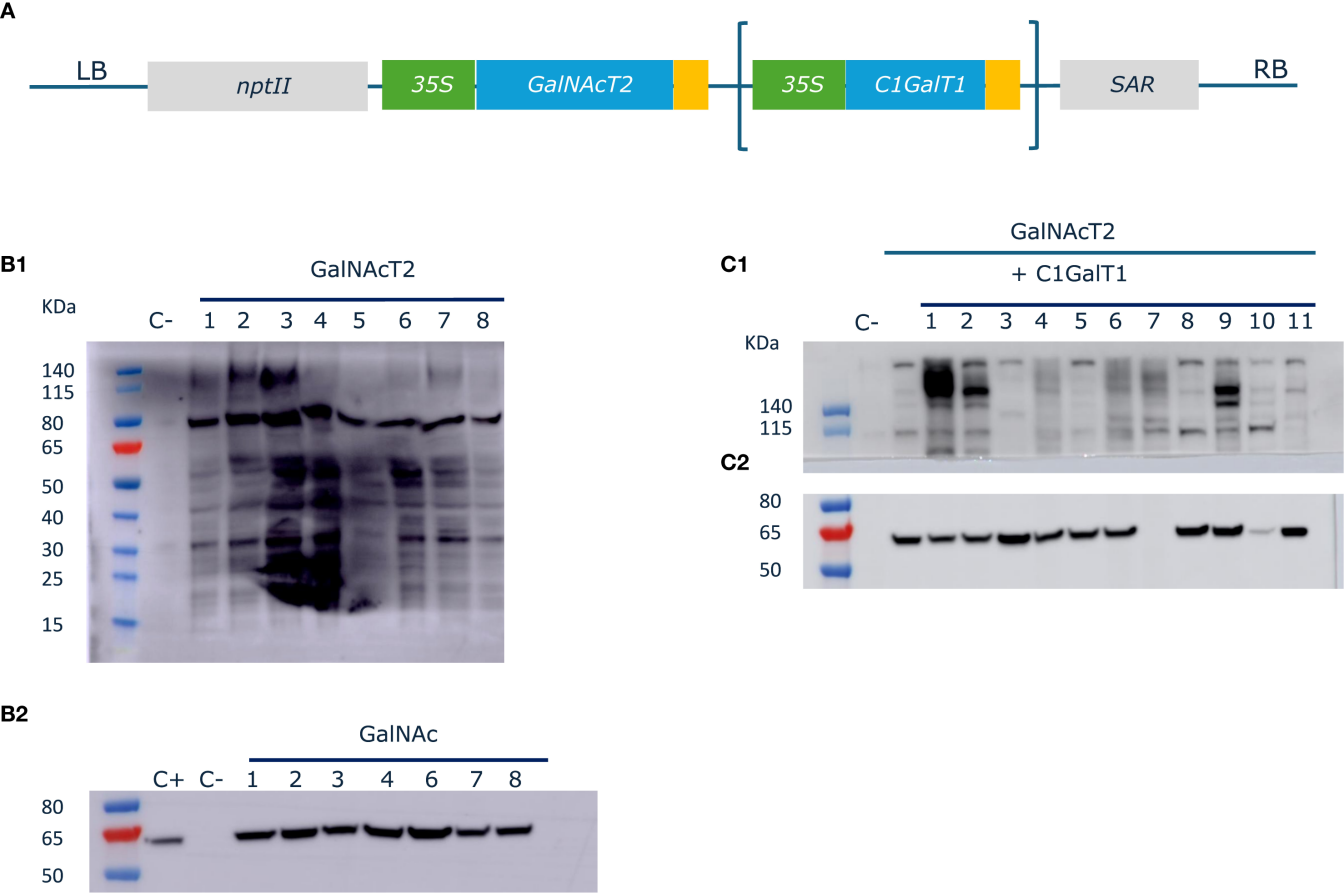
GalNAc-O-glycosylation in BY-2 cells. **(A)**. Schematic overview of the O-glycosylation machinery constructs. The coding sequences for human GalNAc-transferase GalNAcT2 and *D*. *melanogaster* core 1 beta(1,3)-galactosyltransferase C1GalT1 were cloned under the control of *p35S* and *tnos* in level 1 Golden Gate vectors. The expression cassettes were assembled with the *nptII* kanamycin resistance cassette and a *SAR* genetic insulator to generate two different constructs (*GalNAcT2* alone or *GalNAcT2* + *C1GalT1*). **(B)**. Initiation of GalNAc-O-glycosylation in ΔXT/FT BY-2 cells. **(B1)** Twenty-five µg TSP were analyzed by biotinylated *H*. *pomatia* lectin blotting followed by detection HRP-labeled Streptavidin. **(B2)** Twenty-five µg of microsomal fractions were analyzed by western blotting using anti-GalNAcT2 antibodies. A sample derived from the parental ΔXT/FT BY-2 cell line was loaded as a negative control. A sample corresponding to *N. benthamiana* leaves agroinfiltrated with the construct GalNAcT2 alone was used as a positive control (C+). **(C)**. Generation of core 1 structures in ΔXTFT BY-2 cells. **(C1)** Twenty-five µg TSP of transgenic cell lines were analyzed by biotinylated peanut agglutinin blotting followed by detection HRP-labeled Streptavidin. **(C2)** Twenty-five µg of microsomal fractions were analyzed by western blotting using anti-GalNAcT2 antibodies. A sample derived from the parental ΔXTFT BY-2 cell line (C-) or a GalNAcT2 transgenic cell line were used as controls.

The ORFs encoding human GalNAcT2 and *D. melanogaster* C1GalT1 were cloned under the control of the *35S* promotor. The two constructs were assembled into binary vectors to generate two different plasmids encoding either GalNAcT2 alone (GalNAc) or both GalNAcT2 and C1GalT1 (Core1) (**Figure 1A**). The humanized ΔXT/FT BY-2 cell line, which shows the absence of beta(1,2)-xylose and core alpha(1,3)-fucose in *N*-glycans (Mercx et al., 2017), was transformed with the two constructs and several transgenic BY-2 cell lines were selected.

First, the initiation of GaNAc-O-glycosylation pathway was introduced in ΔXT/FT BY-2 cells by expressing the human *GalNAcT2*. We tested GalNAc attachment to endogenous proteins. Total soluble proteins were analyzed by lectin-probed western blotting using *H. pomatia* Agglutinin (HPA), which binds to terminal alpha- and beta-GalNAc residues. A clear HPA signal in all the GalNAc lines was observed compared to the parental ΔXT/FT BY-2 line (first lane in each panel) (**Figure 1B1**). Western blotting of the microsomal fraction of the cell lines probed with anti-GalNAcT2 antibodies confirmed *hGalNACT2* expression in all the GalNAc cell lines tested (**Figure 1B2**).

Elongation of the O-glycans in the core 1 structure was achieved by co-expression of *hGalNAcT2* and *DmC1GalT1* in ΔXT/FT BY-2 cells. Lectin blots of TSPs were probed with peanut (*Arachis hypogaea*) agglutinin (PNA), which has activity toward unsubstituted core 1 (**Figure 1C1**). Strong, albeit highly variable, signals were observed when *DmC1GalT1* was expressed (the membrane was cut due to non-specific signals). The expression of *hGalNAcT2* was verified by western blotting (**Figure 1C2**).

### 3.2 Expression of IgA1 in BY-2 cells expressing mucin-type O-glycosylation enzymes

**Figure 2:**
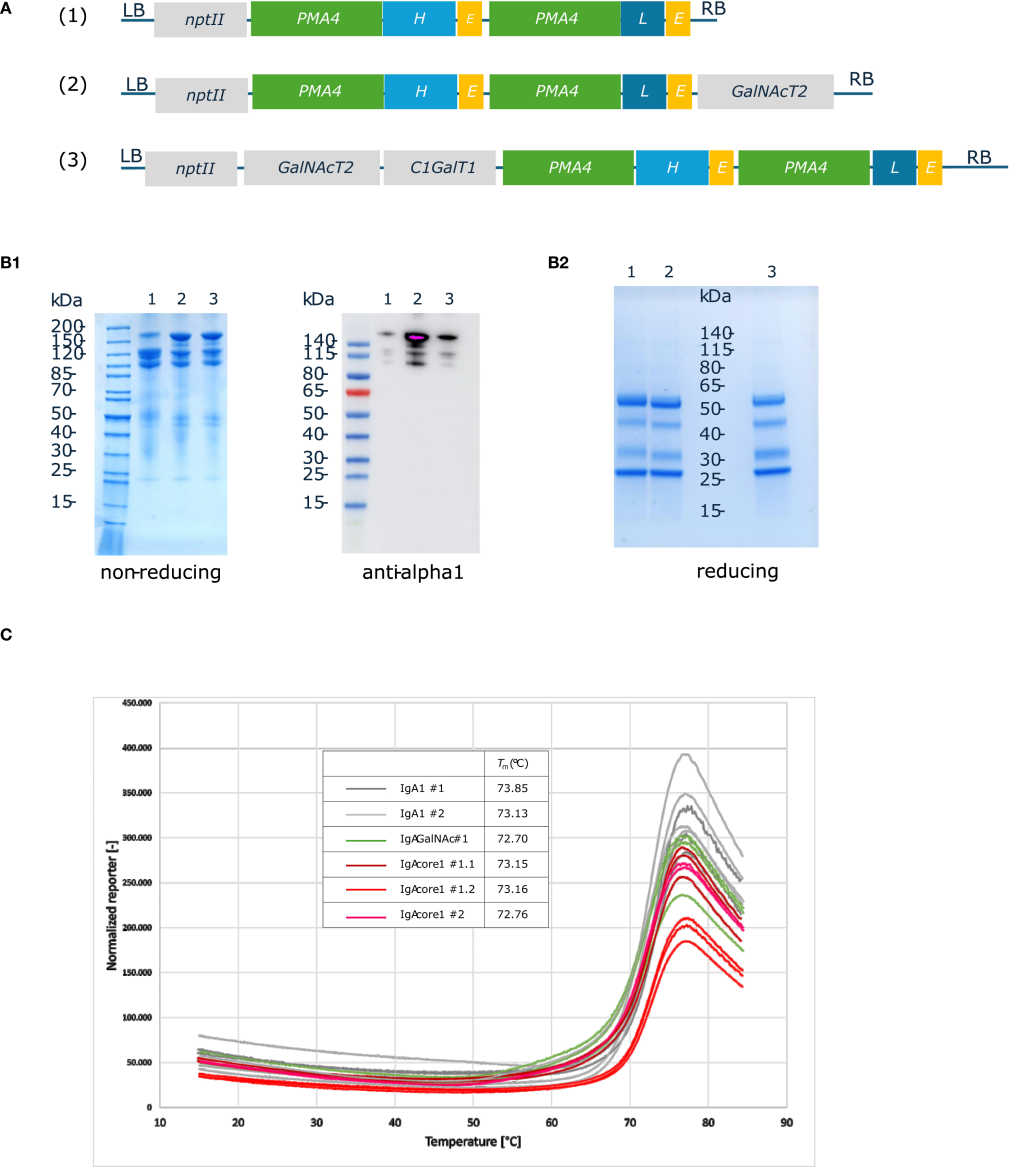
Production and purification of human IgA1 O-glycovariants in BY-2 cells. **(A)**. IgA1 O-glycovariants constructs. The coding sequences from chimeric human Trastuzumab IgA1 HC and LC were cloned under the control of *pPMA4* and *tExtensin* in Level 1 Golden Gate vectors. The expression cassettes were assembled with the O-glycosyltransferase expression cassettes (*GalNAcT2* alone or *GalNAcT2* + *C1GalT1*) and the *npt*II kanamycin resistance cassette to generate three different constructs (*IgA1*, *IgA1 GalNAc* alone or *IgA1 core 1*). **(B)**. SDS-PAGE and immunoblotting of protein A-purified IgA1 O-glycovariants: 1 (IgA1); 2 (IgA1 + GalNAc); 3 (IgA1 core1). Purified samples were analyzed by Coomassie blue staining (2.5 µg) or by western blotting with anti-human IgA (alpha heavy chain) (1: 0.2 µg; 2: 0.4 µg; 3: 0.3 µg). **(C)**. Thermal stability of IgA1 O-glycovariants measured using a thermal shift assay. Each O-glycovariants (two biological replicates #1, #2) was measured in triplicates at temperatures ranging from 15 °C to 85 °C. Melting temperatures were calculated based on the first derivative of the melting curves.

The genes encoding the heavy and light chains of the chimeric human IgA1 with the variable region from Trastuzumab were cloned under the control of the engineered *Nicotiana plumbaginifolia PMA4* promoter and combined with *GalNAcT2* and *C1GalT1* expression cassettes in binary plasmids. A total of three constructs were generated: *IgA1* alone (IgA1), *IgA1* + *GalNAcT2* (IgA1G) and *IgA1* + *GalNAcT2* + *C1GalT1* (IgA1C) (**Figure 2A**). The three *IgA1* constructs were used to transform ΔXT/FT BY-2 cells and stable transgenic cell lines were screened by western blotting with human anti-Fab to identify the best IgA1 producing lines in the three formats (data not shown). Extracellular medium was collected from D11b cultures by filtration and used to purify the three IgA1 O-glycovariants by affinity on Protein A agarose beads. The purified IgA1 O-glycovariants were analyzed using SDS-PAGE (**Figure 2B1**). Under non-reducing conditions, IgA1, IgA1G and IgA1C showed a band at a molecular mass of approximately 160 kDa corresponding to the fully assembled monomeric molecule, as well as two additional bands at 120 kDa and 100 kDa, a pattern similar to that of the same IgA1 antibody expressed in *N. benthamiana* leaves (Göritzer et al., 2017). These three bands were detected by anti-human IgA (heavy chain). Reducing SDS-PAGE confirmed the presence of the alpha heavy chain and the kappa light chain. Two additional bands that could correspond to proteolytic degradation products of the alpha chain were observed (**Figure 2B2**).

The thermal stability of all three IgA1 O-glycovariants was determined, but no significant differences in melting temperatures were observed (**Figure 2C**). The Tm value was approximately 73 °C for the three samples, a value similar to what was observed for other IgA1s produced in *N. benthamiana* leaves (Uetz et al., 2024).

### 3.3 Characterization of the *N*-glycosylation status of the alpha chain

IgA1 is highly glycosylated and characterized by its long 19 residues hinge region, which is GalNAc-O-glycosylated with three to six (Thr225, Thr228, Ser232 and Ser230, Thr233, Thr236) sialylated core 1 O-glycans in human serum (Ohyama et al., 2020). IgA1 also contains two N-glycosylation sites: N263 located in the CH2 and N459 in the tail piece (Göritzer et al., 2020).

**Figure 3:**
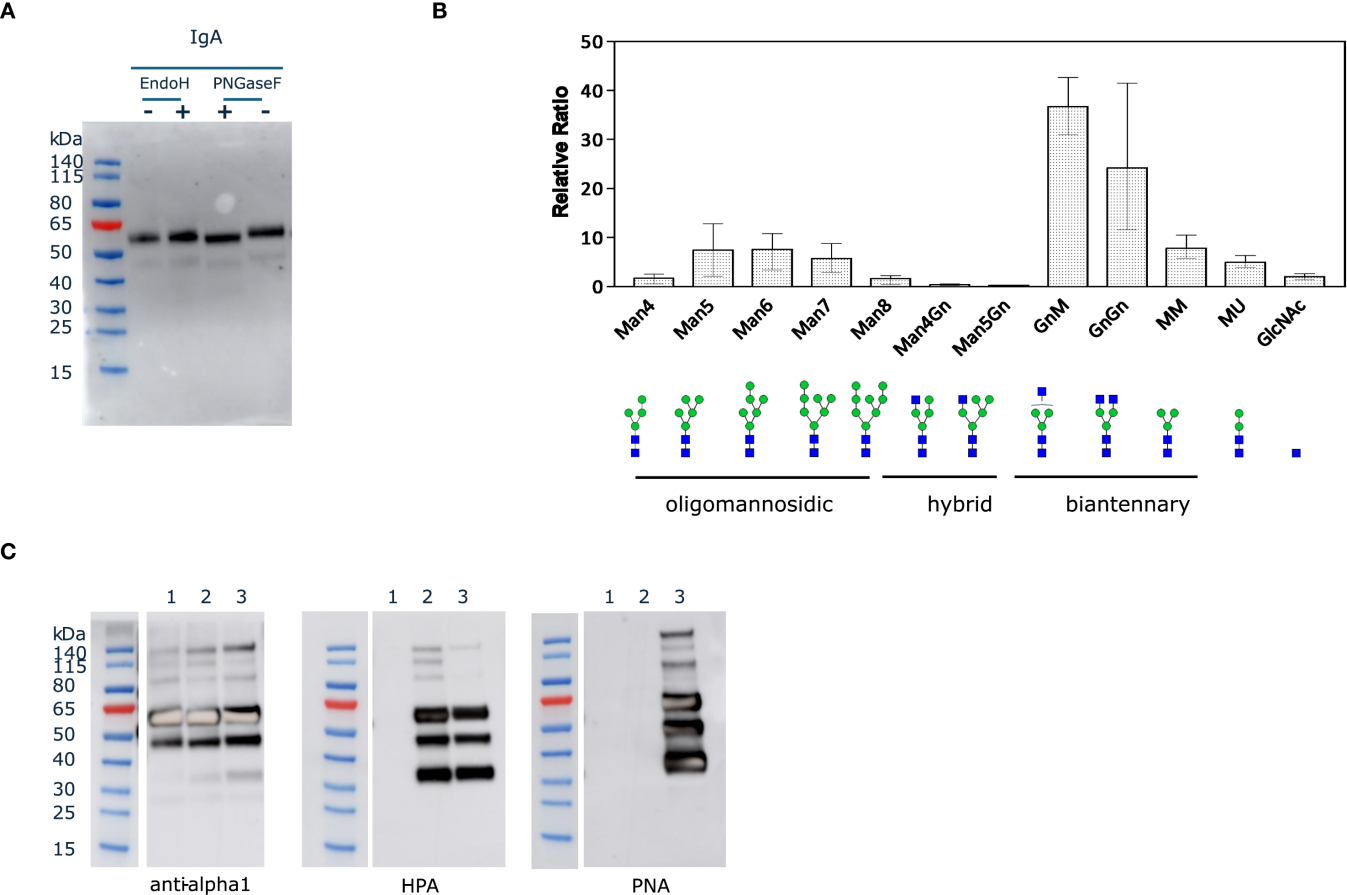
N- and O-glycosylation status of IgA1 in ΔXT/FT BY-2 cells. **(A)**. Purified IgA1 (125 ng) was denatured for 10 min at 100 °C and treated for 1h at 37 °C with EndoH (50 U) or PNGaseF (50 U). Control samples (-) were run in parallel but without the glycosidase. **(B)**. Quantification of the relative abundance of glycoforms corresponding to the glycopeptide ANLTCTLTGLR from the CH2 domain detected on the three IgA1 O-glycovariants. N-glycans are abbreviated according to ProGlycAn system (Altmann et al., 2024). **(C)**. Purified IgA1 O-glycovariants (1.25 µg) were analyzed under reducing conditions by western blotting with anti-human IgA (alpha heavy chain) or lectin blotting with HPA and PNA (1: IgA1; 2: IgA1 + GalNAc; 3: IgA1 core1).

We first assessed the N-glycosylation status of purified IgA1 produced in ΔXT/FT BY-2 cells by *in vitro* deglycosylation with PNGaseF and EndoH. EndoH cleaves high-mannose N-glycans while PNGaseF can cleave all glycan types except those containing core alpha(1,3)-fucose. A slight molecular mass shift of the alpha heavy chain was observed by western blotting in samples treated with PNGaseF, but not with EndoH (**Figure 3A**), indicating that the N-glycans are complex or hybrid types lacking core alpha(1,3)-fucose. This type of profile was previously observed for the alpha chain of sIgA1 purified from ΔXT/FT *N. benthamiana* leaves (Dicker et al., 2016).

Then, the identity of the N-glycans was determined accurately by LC-MS/MS after trypsin and GluC digestion of the purified IgA1 O-glycovariants. The mass spectra of glycopeptides corresponding to the N263 site in CH2 (ANLTCTLTGLR) are shown in **Figure 3B**. The overall profile was broadly similar for the three O-glycovariants, with biantennary complex-type structures like GlcNAc2Man3GlcNAc2 (GnGn) and GlcNAc1Man3GlcNAc2 (GnM/MGn) being the most abundant N-glycans (50-75%), in agreement with the *in vitro* N-deglycosylation assay. The other N-glycans detected were different oligomannosidic (Man4 to Man8) and truncated N-glycans Man3GlcNAc2 (MM). The spectra also showed a small amount of Man2GlcNAc2 (MU) (± 5%) and single Gn (<3%) glycans. No ANLTCTLTGLR peptide without N-glycan was detected, suggesting that N263 was fully occupied.

Glycosylation analysis of the tail piece site (LAGKPTHVNVSVVMAE) yielded poor quality results. This glycosylation site is located at the C-terminus of the protein in a disordered region and was found to be cleaved and underglycosylated for IgA1 expressed in *N. benthamiana* (Dicker et al., 2016; Göritzer et al., 2020).

### 3.4 O-Glycan analysis of the IgA1 hinge region

To assess the identity of the glycans on each IgA1 O-glycovariant, blots of purified IgA1 were probed using anti-human IgA (alpha heavy chain) antibody, as well as HPA and PNA lectins (**Figure 3C**). The same bands were detected with the lectins and the anti-alpha chain antibody, suggesting that O-glycans were found on the HC, probably in the hinge region. The lectin blots clearly indicated that IgA1 was O-glycosylated with mucin-type O-glycans when the appropriate enzymes were co-expressed in ΔXT/FT BY-2 cells. The PNA-probe blot showed that co-expression of *hGalNAcT2* and *DmC1GalT1* was necessary for detection of core 1 O-glycans on alpha chain of IgA1C. In addition, the HPA-probe blot showed that expression of *hGalNAcT2* was necessary to detect GalNAc residues on alpha chain of IgA1G and that both GalNAc and core 1 O-glycans could coexist on IgA1C (when both *hGalNAcT2* and *DmC1GalT1* were expressed), as both HPA and PNA signals were observed for IgA1C (**Figure 3C**). Alternatively, the signals could be due to a small reactivity of HPA toward core 1 glycans as previously reported (Sanchez et al., 2006).

Mass spectrometry analysis was then carried out on the O-glycan containing glycopeptides corresponding to the hinge region (HYTNPSQDVTVPCPVPSTPPTPSPSTPPTPSPSCCHPR). The PTM search included HexNAc and Hex for GalNAc-O-glycosylation as well as Pro hydroxylation and pentoses.

**Figure 4:**
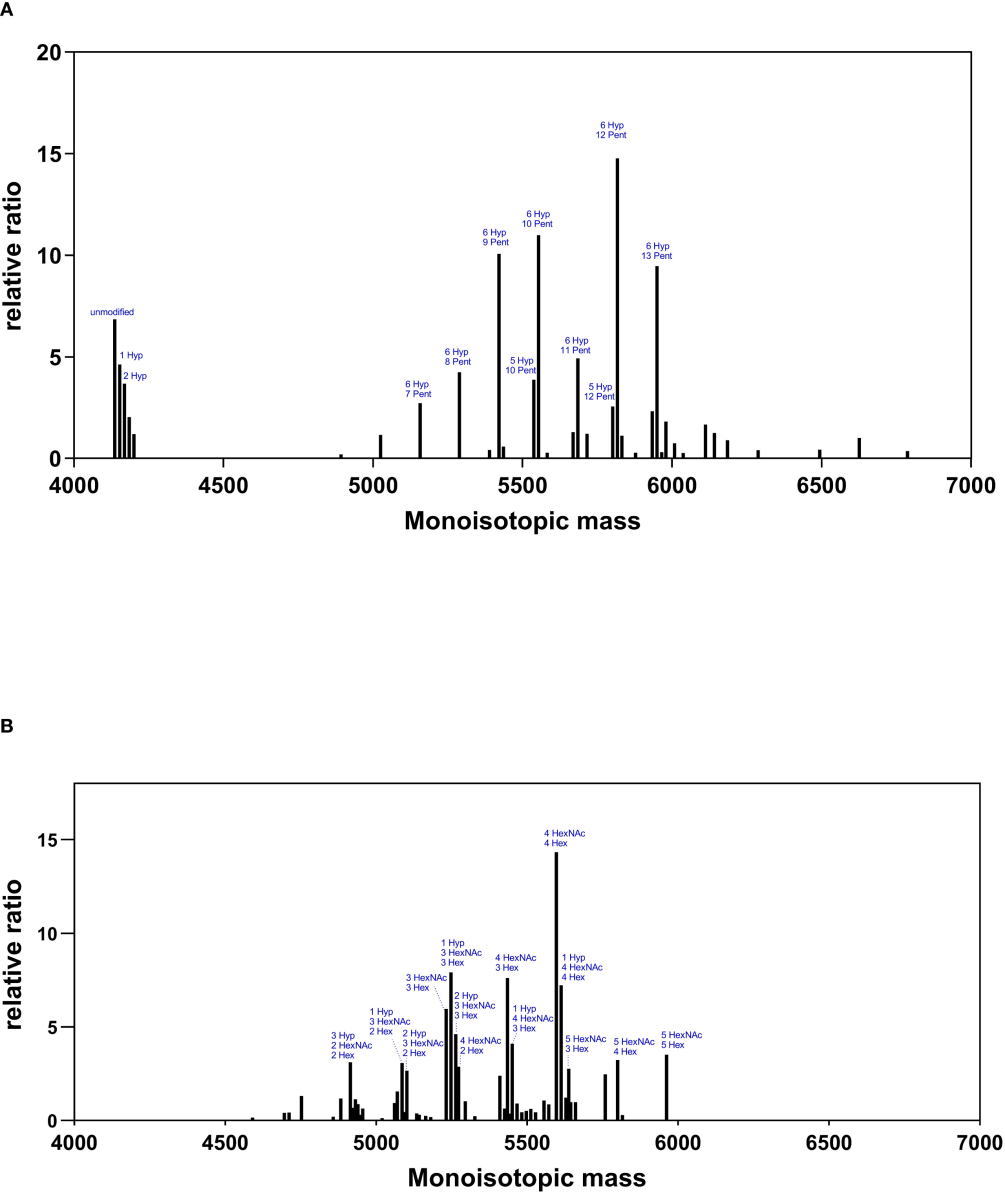
O-glycosylation profiles of IgA1 O-glycovariants in BY-2 cells. Mass spectra of the hinge region peptide (HYTNPSQDVTVPCPVPSTPPTPSPSTPPTPSPSCCHPR) for IgA1 (Panel **(A)**) and IgA1C (Panel **(B)**). Glycosylated peaks with a relative abundance >2.5 are indicated: pentoses (Pent), hydroxyproline (Hyp), N-acetylgalactosamine (HexNAc), and hexoses (Hex).

**Figure 4** and **Supplementary Table 1** show the mass spectra and relative intensities of two different IgA1 O-glycovariants hinge regions. When IgA1 was expressed alone in ΔXT/FT BY-2 cells, the hinge region was heavily modified with plant-specific hydroxyproline glycosylation (**Figure 4A**). Indeed, it was found that more than 90% of the peptide was modified with at least one Hyp and 80% were glycosylated with up to 18 pentoses, with the most abundant glycopeptides being 6 Hyp-9, 10, 12, and 13 Pentoses.

When *hGalNAcT2* and *DmC1GalT1*were co-expressed in ΔXT/FT BY-2 cells, mass shifts corresponding to the addition of HexNAc and Hex were detected, supporting the initiation and elongation of GalNAc-O-glycosylation on the hinge region of IgA1 (**Figure 4B**). Three to five O-sites were occupied, in accordance with the glycosylation status of the same IgA1 observed in HEK 293 (Göritzer et al., 2017). In addition, all identified peptides were modified and showed a strong reduction of Pro hydroxylation and a nearly complete disappearance of pentoses.

## 4 Discussion

We have engineered mucin-type O-glycosylation in *N. tabacum* BY-2 cells. To our knowledge, there is only one example of introducing the first GalNAc step of mucin-type O-glycosylation in transgenic BY-2 cells (Yang et al., 2012a). In this report, a 2A-linked polycistronic construct composed of the human GalNAcT2 and the cytoplasmic Glc (NAc) C4-epimerase from *Pseudomonas aeruginosa* was used because previous data of transient expression in *N. benthamiana* leaves showed no detectable O-glycosylation of the MUC1-YFP reporter when only *hGalNAcT2* was expressed (Yang et al., 2012b). It was indeed postulated that UDP-GalNAc concentration in the Golgi apparatus could not be sufficient to promote efficient GalNAc O-glycosylation of proteins expressed at high levels in *N. benthamiana* leaves (Daskalova et al., 2010). However, additional studies in *N. benthamiana* leaves showed that transient expression of *hGalNAcT2* alone was sufficient to achieve efficient GalNAc O-glycosylation of EPO-Fc (Castilho et al., 2012; Kriechbaum et al., 2020) or human IgA1 (Dicker et al., 2016; Uetz et al., 2024). Our results demonstrated that hGalNAcT2 is sufficient when expressed alone to initiate GalNAc O-glycosylation of endogenous proteins as well as recombinant human IgA1 hinge region in transgenic ΔXT/FT BY-2 cells. However, it is unclear whether heavily glycosylated substrates such as MUC16 (30 sites) would benefit from the co-expression of a Glc (NAc) C4-epimerase.

The elongation of GalNAc-O-glycan to core 1 in yeast (Amano et al., 2008) and *N. benthamiana* leaves (Castilho et al., 2012) was achieved by co-expressing *hGalNAcT2* with *DmC1GalT1*, a core 1 beta1–3 galactosyltransferase which does not require the presence of a specific chaperone COSMC. We used the same approach in transgenic ΔXT/FT BY-2 cells and confirmed the presence of core 1 O-glycan structure on BY-2 cells endogenous proteins and the human IgA1 hinge region. We detected glycopeptides containing 3, 4 and 5 core 1 O-glycan structures and glycopeptides containing both core 1 and Tn O-glycans. The formation of the core 1 O-glycans appeared to be highly efficient with most of the GalNAc being elongated with galactose (0.93 Hex/HexNAc residue ratio). Interestingly, unlike the O-glycosylation analysis of hinge IgA1 produced in *N. benthamiana* leaves co-expressing the core 1 O-glycosylation pathway (Uetz et al., 2024; Dicker et al., 2016), we detected no unmodified hinge glycopeptide, suggesting that GalNAc-O glycosylation was very efficient when *hGalNAcT2* and *DmC1GalT1* were constitutively expressed in transgenic BY-2 cells. The presence of unmodified IgA1 hinge observed in *N. benthamiana* leaves could be related to the high expression of IgA1 under transient expression or to the fact that IgA1C was purified from extracellular medium of BY-2 cells, *i.e.* at the very last step of the secretion process. It is likely that the IgA1C protein sample purified from BY-2 extracellular medium was more homogenous than the IgA1 protein sample from total leaf extracts, which consists of a mixture of IgA1 proteins from the entire secretory pathway (Dicker et al., 2016; Uetz et al., 2024). The use of transgenic BY-2 suspension cultures also facilitates downstream processing by starting from the extracellular medium and offers the possibility of tailoring the culture medium composition, which could complement genetic O-glycoengineering.

In addition, the modification of the hinge region with mucin-type O-glycans resulted in a strong reduction in the abundance of Hyps in the hinge region, suggesting that Pro hydroxylation was somehow prevented. Nevertheless, Pro hydroxylation and further Hyp glycosylation still occurred and may be deleterious for the properties of the antibody. Aberrant Hyp glycosylation was also detected on the hinge region of the same IgA1 produced in *N. benthamiana* leaves with glycopeptides containing 5 hydroxyprolines with 4 to 10 pentoses (Göritzer et al., 2017; Mócsai et al., 2021). Similarly, other IgA1 produced in *N. benthamiana* leaves revealed a similar O-glycosylation status with the presence of glycopeptides containing up to 6 hydroxyprolines with 8 and 10 pentoses (Karnoup et al., 2005; Dicker et al., 2016) or 5 hydroxyprolines and 4 to 10 pentoses (Uetz et al., 2024, Uetz et al., 2022; Göritzer et al., 2020). A promising strategy to reduce this plant-specific Hyp glycosylation is the silencing of P4Hs, the enzymes responsible for Pro hydroxylation, a strategy being explored in *N. benthamiana* leaves (Uetz et al., 2022, Uetz et al., 2024; Mócsai et al., 2021).

## Data Availability

The original contributions presented in the study are included in the article/**Supplementary Material**. Further inquiries can be directed to the corresponding authors.
