# Supplementary material for: Production of recombinant IgA1 with defined mucin-type O-glycans in Nicotiana tabacum BY-2 cells
Source: Front Plant Sci. 2025 Sep 24;16:1675517. doi: 10.3389/fpls.2025.1675517 (PMC12504281; doi:10.3389/fpls.2025.1675517)
Supplement: Supplementary file 1 [file DataSheet1.pdf]

Supplementary Table 1

Quantification of hinge region O-glycans of recombinantly produced BY-2 IgA1 and IgA1 core 1.

IgA1		
Monoisotopic.Mass	Glycoform	Relative Abundance
4136.889077	0xHyp0xPent0xHexNAc0xHex	6.8
4152.880377	1xHyp0xPent0xHexNAc0xHex	4.6
4168.877777	2xHyp0xPent0xHexNAc0xHex	3.7
4184.872277	3xHyp0xPent0xHexNAc0xHex	2.0
4200.868277	4xHyp0xPent0xHexNAc0xHex	1.2
4893.078577	6xHyp5xPent0xHexNAc0xHex	0.2
5025.116077	6xHyp6xPent0xHexNAc0xHex	1.2
5157.155577	6xHyp7xPent0xHexNAc0xHex	2.7
5289.192577	6xHyp8xPent0xHexNAc0xHex	4.2
5389.246677	4xHyp9xPent0xHexNAc0xHex	0.4
5421.233577	6xHyp9xPent0xHexNAc0xHex	10.1
5436.244377	7xHyp5xPent1xHexNAc2xHex	0.6
5537.281977	5xHyp10xPent0xHexNAc0xHex	3.9
5553.280277	6xHyp10xPent0xHexNAc0xHex	11.0
5582.296977	6xHyp5xPent1xHexNAc3xHex	0.3
5669.317677	5xHyp11xPent0xHexNAc0xHex	1.3
5685.322077	6xHyp11xPent0xHexNAc0xHex	4.9
5715.341777	6xHyp10xPent0xHexNAc1xHex	1.2
5801.362377	5xHyp12xPent0xHexNAc0xHex	2.6
5817.368177	6xHyp12xPent0xHexNAc0xHex	14.8
5832.368577	7xHyp8xPent1xHexNAc2xHex	1.1
5877.397077	6xHyp10xPent0xHexNAc2xHex	0.3
5933.424877	5xHyp13xPent0xHexNAc0xHex	2.3
5949.400777	6xHyp13xPent0xHexNAc0xHex	9.5
5965.405877	7xHyp13xPent0xHexNAc0xHex	0.3
5979.406677	6xHyp12xPent0xHexNAc1xHex	1.8
6008.437877	6xHyp7xPent1xHexNAc4xHex	0.8
6037.446577	4xHyp9xPent0xHexNAc4xHex	0.3
6111.467777	6xHyp13xPent0xHexNAc1xHex	1.7
6141.473077	6xHyp12xPent0xHexNAc2xHex	1.3
6185.483677	5xHyp10xPent0xHexNAc4xHex	0.9
6287.522477	5xHyp12xPent0xHexNAc3xHex	0.4
6493.572077	7xHyp17xPent0xHexNAc0xHex	0.4
6625.611277	7xHyp18xPent0xHexNAc0xHex	1.0
6786.667377	7xHyp14xPent1xHexNAc3xHex	0.4

IgA1 core 1		
Monoisotopic.Mass	Glycoform	Relative Abundance
4591.030177	3xHyp0xPent2xHexNAc0xHex	0.2
4696.062077	2xHyp0xPent1xHexNAc2xHex	0.4
4712.060077	3xHyp0xPent1xHexNAc2xHex	0.4
4753.085277	3xHyp0xPent2xHexNAc1xHex	1.3
4858.112477	2xHyp0xPent1xHexNAc3xHex	0.2
4883.140577	1xHyp0xPent2xHexNAc2xHex	1.2
4915.134477	3xHyp0xPent2xHexNAc2xHex	3.1
4924.167577	1xHyp0xPent3xHexNAc1xHex	0.7
4931.146077	4xHyp0xPent2xHexNAc2xHex	1.1
4940.168377	2xHyp0xPent3xHexNAc1xHex	0.9
4947.137877	5xHyp0xPent2xHexNAc2xHex	0.3
4956.162177	3xHyp0xPent3xHexNAc1xHex	0.6
5020.168277	2xHyp0xPent1xHexNAc4xHex	0.1
5061.199077	2xHyp0xPent2xHexNAc3xHex	0.9
5070.229477	0xHyp0xPent3xHexNAc2xHex	1.6
5086.224177	1xHyp0xPent3xHexNAc2xHex	3.1
5092.196877	2xHyp3xPent1xHexNAc2xHex	0.5
5102.224777	2xHyp0xPent3xHexNAc2xHex	2.7
5134.214477	4xHyp0xPent3xHexNAc2xHex	0.4
5143.239377	2xHyp0xPent4xHexNAc1xHex	0.3
5164.224477	2xHyp6xPent1xHexNAc0xHex	0.2
5181.227077	0xHyp3xPent0xHexNAc4xHex	0.2
5232.290577	0xHyp0xPent3xHexNAc3xHex	6.0
5248.278677	1xHyp0xPent3xHexNAc3xHex	7.9
5264.273577	2xHyp0xPent3xHexNAc3xHex	4.6
5273.310877	0xHyp0xPent4xHexNAc2xHex	2.9
5295.270977	2xHyp3xPent2xHexNAc2xHex	1.0
5327.262777	4xHyp3xPent2xHexNAc2xHex	0.2
5410.332077	1xHyp0xPent3xHexNAc4xHex	2.4
5426.323977	2xHyp0xPent3xHexNAc4xHex	0.6
5435.360177	0xHyp0xPent4xHexNAc3xHex	7.6
5441.323077	1xHyp3xPent2xHexNAc3xHex	0.4
5451.358377	1xHyp0xPent4xHexNAc3xHex	4.1
5467.353677	2xHyp0xPent4xHexNAc3xHex	0.9
5482.354777	1xHyp3xPent3xHexNAc2xHex	0.4
5497.366377	0xHyp6xPent2xHexNAc1xHex	0.5
5512.356177	1xHyp2xPent3xHexNAc3xHex	0.6
5528.357377	2xHyp2xPent3xHexNAc3xHex	0.4
5556.383977	0xHyp0xPent3xHexNAc5xHex	1.1
5572.380777	1xHyp0xPent3xHexNAc5xHex	0.9
5597.420577	0xHyp0xPent4xHexNAc4xHex	14.3
5613.416277	1xHyp0xPent4xHexNAc4xHex	7.2
5629.400477	2xHyp0xPent4xHexNAc4xHex	1.2
5638.450177	0xHyp0xPent5xHexNAc3xHex	2.8
5644.412477	1xHyp3xPent3xHexNAc3xHex	1.0
5660.404277	2xHyp3xPent3xHexNAc3xHex	1.0
5759.470477	0xHyp0xPent4xHexNAc5xHex	2.5
5800.506077	0xHyp0xPent5xHexNAc4xHex	3.2
5816.481877	1xHyp0xPent5xHexNAc4xHex	0.3
5962.549477	0xHyp0xPent5xHexNAc5xHex	3.5
